# Integrating parallel *Plasmodium falciparum* and *Plasmodium vivax* malaria models in a unified framework to capture co-endemic prevalence patterns

**DOI:** 10.1038/s44528-026-00007-4

**Published:** 2026-07-07

**Authors:** Richard J. Sheppard, Giovanni D. Charles, Constanze Ciavarella, Nora Schmit, Shazia N. Ruybal-Pesántez, Gina Cuomo-Dannenburg, Tom R. Brewer, Michael T. White, Peter Winskill

**Affiliations:** 1 MRC Centre for Global Infectious Disease Analysis, Department of Infectious Disease Epidemiology, Imperial College, London, UK; 2 PCCEI, Université de Montpellier, INSERM, Université des Antilles, Montpellier, France; 3 Instituto de Microbiología, Universidad San Francisco de Quito, Quito, Ecuador; 4 Pandemic Sciences Institute, Nuffield Department of Medicine, University of Oxford, Oxford, United Kingdom; 5 Infectious Disease Epidemiology and Analytics G5 Unit, Department of Global Health, Institut Pasteur, Université Paris-Cité, IN SERM U1347, Paris, France

**Keywords:** Diseases, Ecology, Ecology, Microbiology

## Abstract

**Background:**

*Plasmodium falciparum* and *Plasmodium vivax* cause most malaria cases worldwide and are co-endemic in many countries, yet differ substantially in biology and in their responses to common interventions. As programmes drive down *P. falciparum*, *P. vivax* is a growing challenge for elimination, but most modelling tools assess the species separately, limiting coordinated policy. We aimed to build a unified malaria transmission modelling framework for co-endemic settings and assess how well it reflects global prevalence.

**Methods:**

We integrated an established *P. vivax* model into a flexible *P. falciparum* modelling platform, enabling parallel simulation of both species within a shared biological, demographic, and intervention environment. Modelled equilibrium prevalences, matched by mosquito density, were compared with 19,225 yearly co-prevalence estimates from the Malaria Atlas Project (769 sub-national regions, 33 co-endemic countries, 2000–2024); uncertainty was represented by 95% quantile-based regions from 50 parameter draws. We assessed how this fit was modified by biological factors and simulated interventions.

**Results:**

Here we show that the framework captures 51% of co-prevalence estimates within its uncertainty regions, rising to 65.5% when country-specific *P. vivax* relapse rates and human Duffy negativity are included. *P. falciparum* predominates where mosquito densities are high, whereas *P. vivax* is relatively more prevalent at lower densities. Simulated interventions produce larger relative reductions in *P. falciparum* prevalence, while *P. vivax* shows greater rebounds after intervention withdrawal, particularly at low mosquito densities.

**Conclusions:**

This unified framework provides a quantitative tool to support coordinated, species-specific intervention strategies in co-endemic settings, a step toward sustainable malaria elimination.

## Introduction

Malaria remains a major global health challenge, with *Plasmodium falciparum* and *Plasmodium vivax* responsible for most clinical cases worldwide^1,2^. While *P. falciparum* is the predominant malaria species across sub-Saharan Africa, and contributes the highest number of clinical cases and deaths worldwide^2^, *P. vivax* tends to have a similar or higher prevalence in malaria-endemic regions outside Africa (Asia and the Americas), where it frequently becomes the primary target of elimination efforts^3^.

The biology of *P. vivax* differs from *P. falciparum* in several important ways that affect transmission and control. Following human infection via an infective mosquito bite and sporozoite migration to the liver, *P. vivax* parasites form a reservoir of dormant hypnozoite cells that can reactivate at a later date, causing relapse infections weeks or months after the initial blood-stage infection has been resolved^4^. While blood-stage infections for either parasite can be cleared by schizont-targeting drugs such as artemisinin combination therapies or chloroquine^1^, *P. vivax* infections require additional treatment with 8-aminoquinoline drugs (primaquine and tafenoquine) for hypnozoite clearance^5^. 8-aminoquinolines carry risks of haemolysis in individuals with glucose-6-phosphate dehydrogenase (G6PD) deficiency^6^, and their use requires G6PD testing before administration to minimise the adverse effects of these drugs^7–10^.

A major factor that limits *P. vivax* transmission across most of Africa is the high prevalence of the human Duffy-negative blood group phenotype, which confers protection against the invasion of erythrocytes by *P. vivax* parasites^11^. Correspondingly, areas within Africa that have higher *P. vivax* burden are those where Duffy negativity is less prevalent^12^, such as Ethiopia and Madagascar^2^. In recent years, however, there has been an increase in identification of *P. vivax* at low levels in high Duffy prevalence countries, facilitated by the increased use of highly sensitive diagnostic tests (e.g., quantitative polymerase chain reaction (PCR)) to detect infections with low parasitaemia^13–16^. These observations have led to speculation and investigation into molecular mechanisms that allow erythrocyte invasion in Duffy-negative individuals^17,18^. It is likely that Duffy negativity provides strong but incomplete protection from *P. vivax* infection and that the parasite may be endemic in more countries than is commonly thought^19^.

The estimation of true *P. vivax* prevalence may be further complicated by the incorrect parasite species identification of *Plasmodium* infections by light microscopy (LM). There is evidence that some monoparasitic *P. falciparum* and *P. vivax* infections are misidentified as the other and that some mixed *P. falciparum*/*P. vivax* infections are misidentified as monoparasitic infections (sometimes described as masking)^20–22^. Misidentification of monoparasitic infections and masking of mixed infections may occur due to biases in the expectations of microscopists in regions where one parasite dominates above the other^20^, and may be particularly strong against the detection of *P. vivax* infections in areas of high Duffy negativity where only *P. falciparum* is believed to be endemic and regularly tested for^23^. Mixed infections may be further affected by differential species-specific parasitaemia in the blood, where *P. vivax* has comparatively low parasitaemia compared with *P. falciparum*^24,25^, and may therefore be identified less regularly as a result. Misdiagnosis may result in sub-optimal drug selection for treatment of blood-stage infections and neglecting treatment of *P. vivax* infections with anti-hypnozoite drugs^21^. The evidence to quantify the scale and variability of misidentification has not yet been systematically reviewed.

Mathematical models provide a powerful means to represent and compare the contrasting biologies of *P. falciparum* and *P. vivax*, and to investigate how these differences shape transmission dynamics. Modelling efforts have historically focused on *P. falciparum*, given its high level of disease burden compared with *P. vivax*^1^, and have an extensive history of use since the formalisation of the Ross-Macdonald model in 1957^26^. There are now several established *P. falciparum* models that are flexible, generalisable, and customisable, capturing complexity such as human disease status, immunity to malaria and mosquito biting heterogeneity^27–29^. These models are frequently used to assess the prospective effectiveness of new interventions and support National Malaria Control Programme (NMCP) decision-making.

The first *P. vivax* specific model, in contrast, was designed in 1988^30^, and around 50 *P. vivax* transmission models have since been published^31^. Of all *P. vivax* models, a subset model both *P. falciparum* and *P. vivax* parasites in parallel^32–36^, and a smaller subset model interactions between the parasite species, including features such as parasite coinfection, treatments, masking, and a hypothesised mechanism by which *P. falciparum* infections trigger *P. vivax* relapses^34,37–39^. These *P. vivax* models have been used to investigate a range of intervention scenarios across *P. vivax* endemic countries, but are limited by their mathematical abstraction from reality, key parasite biology that they omit, their accessibility or their generalisability and applicability to various contexts.

Given the need for new tools to better understand transmission dynamics in co-endemic settings, there is value in developing models for both species that share a computational framework for biological processes they have in common, while representing species-specific differences explicitly. A key advantage of this approach is being able to configure simulations with aligned geographic scope, vector species, population demography, and intervention histories, enabling consistent comparison of malaria dynamics and impact across both species.

*malariasimulation*^29^ is the most recent iteration of Imperial College London (ICL)’s 2010 individual-based (IB) malaria model^40^; a complex, accessible and extensible malaria transmission model with mosquito and human disease components and that has a corresponding deterministic version^41^. The IB model incorporates mosquito biting heterogeneity, seasonal patterns, infection latency, human immunity and disease severity, and has, to date, been designed to model *P. falciparum*^29^. The package includes a wide range of in-built control interventions and helper tools for model initiation, calibration and costing^42^. This model has been used to assess the impact of malaria control interventions^40,43,44^, provide NMCPs with support and investigate key biological questions^45,46^.

White et al.’s 2018 *P. vivax* model^47^ was designed using ICL’s *P. falciparum* malaria model as a template, following the same broad structure with corresponding deterministic and IB versions, and fitted to data from Papua New Guinea (PNG) and the Solomon Islands, covering a range of transmission settings^47^. It models hypnozoites at the batch level (representing the hypnozoites formed following a single infective bite), balancing biological complexity with computational efficiency, and includes key biological details such as human immunity and mosquito biting heterogeneity following the patterns established in ICL’s malaria model^29,40,41^. It has been used to investigate the impact of interventions including mass drug administration and insecticide-treated net (ITN) distributions^47^, radical cure (the combination of schizont- and hypnozoite-targeting treatment)^8^, G6PD testing^48,49^, serological test-and-treat^9,10^, and vaccination^50,51^. The deterministic version of this model has previously been brought back into ICL’s deterministic malaria model for parallel modelling and used to optimise constrained resources in ITN distributions in co-endemic settings in the pursuit of malaria elimination^39^.

Here, we integrate White et al.’s IB *P. vivax* model into the *malariasimulation* package, creating a unified framework in which both species can be simulated in parallel within a shared biological, demographic, and intervention environment. Comparing matched-mosquito-density model equilibria with global Malaria Atlas Project (MAP) prevalence estimates, the framework reproduces a substantial proportion of observed co-prevalence variation, with capture improving when country-specific Duffy negativity and *P. vivax* relapse rates are incorporated. *P. falciparum* dominates at high mosquito densities, whereas *P. vivax* becomes relatively more prevalent at lower densities and is sustained there by hypnozoite relapses. Simulated interventions produce larger relative reductions in *P. falciparum* than in *P. vivax* prevalence, and *P. vivax* shows greater rebounds after intervention withdrawal, consistent with the resilience conferred by hypnozoite reservoirs. Together, these findings indicate that conventional control tools alone are unlikely to be sufficient for *P. vivax* in elimination settings, and that the integrated framework provides a practical platform for evaluating species-specific intervention strategies, supporting coordinated decision-making by NMCPs, and advancing research on inter-parasite interactions, Duffy negativity, and G6PD deficiency.

## Methods

### Global parasite prevalence trends

Malaria prevalence estimates from MAP^52^ were accessed via the *site* R package^53^. LM-detectable prevalences for both parasites are given for each year from 2000 to 2024, where *Plasmodium falciparum* prevalence is estimated for the ages of 2–10 years and *Plasmodium vivax* prevalence is given for all ages (1–99 years) at the admin 1 level (e.g., province), where admin 1 areas are split into urban and rural regions. Urban and rural regions were determined using a population threshold of 1500 people per square km based on estimates from WorldPop^54^ and the World Population Prospects^55^ as described by the *site* R package^53^. Prevalence estimates were first filtered to include all regions (at the admin 1 urban/rural level) that have had positive estimated prevalence of *P. falciparum* and *P. vivax* for at least one timepoint during 2000–2024 (which may be at different years for each parasite), indicating regions where both parasites are or may have been present. This resulted in 34 countries where both parasite species have been present in any year since 2000, comprising 725 admin 1 regions, or 1371 regions at the urban/rural level.

Prevalence estimates were transformed using the logit function for clustering to enhance resolution at lower prevalence levels. As the logit transform is undefined at 0 and 100%, regions with zero prevalence estimates for either species at any time point were excluded. However, this exclusion was not solely driven by the transformation. In many cases, zero values represent structural absence or interruption of transmission, resulting in trajectories dominated by persistent or intermittent zeros. Including such trajectories in a distance-based clustering framework led to cluster structures primarily reflecting presence versus absence, rather than differences in co-endemic transmission dynamics. We therefore restricted the analysis to regions with non-zero prevalence for both species across the study period to enable meaningful comparison of joint temporal patterns. This led to the complete exclusion of 246 admin 1 regions, or 602 regions at the urban/rural level, resulting in 769 paired prevalence trajectories through time where each included trajectory represents a complete set of yearly non-zero prevalence estimates from 2000 to 2024 (Supplementary Fig. 1). Many of these excluded regions describe the elimination of one or both species, while others show more erratic prevalence patterns that feature apparent unstable parasite elimination, likely based on limited data (e.g., regions in Chad, Somalia and South Sudan that show 0% prevalence between higher positive estimates). Several countries were left with only a few trajectories to use in the analysis (those with non-zero prevalence in all years, out of the set of regions where both *P. falciparum* and *P. vivax* were present in any year): Central African Republic (CAR, 2/2), Chad (4/7), Djibouti (4/8), Guatemala (1/43), Honduras (6/35), Kenya (4/8), Panama (3/23), Philippines (1/116) and South Sudan (6/10), while all (20) regions from Haiti were removed. In most cases, the remaining trajectories visibly cover the prevalence space of filtered trajectories, suggesting that the removal of the trajectories would not substantially reflect a loss in qualitative trends captured and examined. Exceptions to this are regions in Djibouti, Ecuador, Panama and Yemen that have higher *P. falciparum* prevalence relative to other trajectories, regions in Kenya that have lower *P. vivax* prevalence than included regions, regions in Honduras that have low *P. falciparum* and high *P. vivax* prevalence compared to other trajectories, and regions in Guatemala, Philippines and Somalia where the few included trends do not reflect all the variation seen.

We grouped these regional co-prevalence trends into historical archetypes, broadly representing the ways that parasite co-prevalences have changed together through time, using a simple *k*-means clustering algorithm in R. The region-specific *P. falciparum* and *P. vivax* prevalence estimates at each year during 2000–2024 were treated as a set of features (25 years × 2 parasite species = 50 features per region) for clustering in the *fviz_nbclust* function from the *factoextra* R package^56^ and optimised using the Gap statistic (method = gap_stat, k.max = 20), which compares the clustering with a null reference distribution to identify meaningful clusters.

Clustered prevalence trajectories were labelled to describe key characteristics (such as malaria burden, parasite ratio and relative change during 2000–2024), then summarised by applying a loess smooth model (using the *loess* function from the *stats R* package^57^ with default inputs) to yearly species-specific population-at-risk weighted mean prevalences for each parasite, where populations-at-risk were estimated using population sizes from WorldPop^54^, filtered to 1 km pixels where malaria prevalence was > 0% as estimated by MAP^52^, as described by the *site* R package^53^.

### Model integration

We implemented an in-house version of White et al.’s 2018 *P. vivax* model^47^ into the existing *malariasimulation* R package, version 2.02^29^. The structure of White et al.’s *P. vivax* model was based on the *malariasimulation* model, with an identical deterministic mosquito model and an analogous IB human model, which accounts for biting heterogeneity, multiple forms of exposure- and age-dependent immunity, and seasonality. The primary difference between the *P. falciparum* and *P. vivax* models is the tracking of the individual-level number of hypnozoite batches in the *P. vivax* model, which increases through new infective bites, decreases at an exponential rate, and can cause relapses that increase in rate with the number of hypnozoite batches. The similarity in model structure made it possible to bring White et al.’s model back into *malariasimulation*, integrating it with the package’s wide malaria intervention toolkit and supplementary packages^42^. The models have further minor conceptual differences, where the *P. falciparum* model has human states based on symptom status (clinical disease, asymptomatic, sub-patent), whereas the *P. vivax* model is delineated by parasitaemia (clinical disease, LM-detectable, PCR-detectable). In addition, some types of immunity are parasite-specific (where the *P. falciparum* model includes immunity to clinical and severe disease, immunity to detection and pre-erythrocytic immunity, and the *P. vivax* model includes immunity to clinical disease and anti-parasite immunity). White et al.’s *P. vivax* model was parameterised through fitting to data from PNG and the Solomon Islands^47^, capturing a range of transmission settings, and we carried these parameter estimates forward into our default *P. vivax malariasimulation* parameter set. Full model details are found in the relevant papers^29,40,47,58^, with a summary of key parasite model differences and adjustments that were made to bring White et al.’s *P. vivax* model more in line with the *malariasimulation* structure and instructions for use available in the *malariasimulation* vignettes^59^.

The models use deterministic equilibrium solutions to generate the initial conditions of the dynamic IB model simulations. For a given initial adult entomological inoculation rate (EIR; the number of infectious mosquito bites per person per year), human population size, mosquito species (or species mix), biting heterogeneity and human treatment information, the equilibria can be used to calculate the human disease states and immunity levels, which are then used to calculate the force of infection on mosquitos (FOIM). The EIR and FOIM are then used together to calculate the baseline total number of modelled mosquitos per person (referred to in this text as the mosquito density). These outputs are then used to initialise the *malariasimulation* variable set used in simulation.

### Model equilibrium comparison analyses

To compare the *P. vivax* and *P. falciparum* models at the same location, we began with the simplifying assumption that transmission of both species is driven by the same underlying mosquito population, with the same mosquito density and species (or species mix). Each parasite model takes an initial EIR as an input to calculate a corresponding equilibrium solution (which includes disease prevalence, FOIM and number of mosquitos per person); the relationships between EIR and these outputs differ between the parasite models. We therefore examine the relationships between EIR, LM- and PCR- detectable prevalence and the total number of mosquitos per person for each parasite model using EIR values between 10^−12^ and 100 infectious bites per person per year. Assuming that parasite prevalences in a region must have a matching mosquito density in both model equilibria, we generated predicted parasite-specific LM- and PCR-detectable prevalences over a range of comparable mosquito densities. We applied a loess fit to the mosquito density-prevalence relationship using the *predict* and *loess* functions in *R* (where span = 0.1).

The main results were calculated using each model’s default parasite-specific parameter set, taken from posterior draws from their respective Markov-chain Monte Carlo model fits^29,47^. To represent the resulting parameter uncertainty, we repeated these calculations over 50 parameter draws per parasite, generating 95% quantiles. For comparisons across the dual prevalence axes, we combined these quantiles in both dimensions to define quantile-based uncertainty regions (QBURs) around the default parameterisation, capturing approximately 90% of the total variation. The prevalence space covered by each QBUR across the range of mosquito densities was summarised using the *geom_mark_hull* function from the *ggforce* package^60^.

Matching the parasite models by mosquito density is a strong but, we argue, not unreasonable assumption: it allows us to assess how well the existing fitted models capture observed co-prevalence variation without introducing additional sources of complexity such as mosquito- and parasite-species-specific differences in transmissibility within mixed mosquito populations. Such effects would need to be considered carefully when tailoring the framework to real-world settings, but lie outside the scope of this analysis.

We compared the matched equilibrium solutions to the global parasite prevalence trends from MAP to assess how much of the observed variation can be explained by the equilibria alone. We then explored how this comparison was modified by a range of biological, methodological and ecological factors, varying each in turn while holding the others at default values:

#### Misidentification of *P. vivax* monoinfections as *P. falciparum*

Observed *P. vivax* and *P. falciparum* prevalences measured using LM and PCR often differ^24,25^, suggesting that *P. vivax* infections can often be misdiagnosed as *P. falciparum*. We investigated the impact of variable misdiagnosis rates on observed LM-detected *P. vivax* and *P. falciparum*, assuming that detection is biased in favour of *P. falciparum* due to relative parasitaemia and expectations from parasite prevalence burden^23^. A fixed proportion (between 10 and 70%) of *P. vivax* prevalence was re-attributed to *P. falciparum*.

#### Masking of *P. vivax* by *P. falciparum* in mixed infections

Mixed *P. falciparum* and *P. vivax* infections may be diagnosed by LM as *P. falciparum* infections, a phenomenon known as masking. We assumed independence of infection by each parasite, so that the prevalence of mixed infections is the product of the two species-specific prevalences. We then assumed, as an upper bound on this effect, that all mixed infections were diagnosed as *P. falciparum*, subtracting the mixed-infection prevalence from observed *P. vivax* prevalence while leaving observed *P. falciparum* prevalence unchanged.

#### Prevalence of Duffy negativity

Duffy negativity provides at least partial protection from *P. vivax* infection and is relevant in locations where both Duffy negativity and *P. vivax* are present at moderate prevalences (e.g., Madagascar, Ethiopia, Sudan). To approximate this, we assumed that a fixed proportion of the population (50, 90, 99%) is fully protected from *P. vivax* infection, with the equilibrium solution applied only to the remaining susceptible proportion. With 90% Duffy negativity, for example, modelled prevalence is reduced by an order of magnitude relative to a fully susceptible population. Country-level Duffy negativity prevalences were obtained from MAP^52^ via the *site* package^53^, with values listed in Supplementary Table 1.

#### Hypnozoite relapse rate

Rates of relapse are known to vary geographically, likely with genetic and seasonal causes. Battle et al.^61^ estimated regional mean times to relapse ranging from 41 days (South East Asia, our default) to 152–164 days (Central America), with intermediate values across Oceania, South America, sub-Saharan Africa, South Asia, and the Eastern Mediterranean. We calculated equilibrium solutions for *P. vivax* at the midpoints of each regional group, with results shown for a representative subset (mean time to relapse of 41, 56, 114 and 158 days). Country-specific relapse rates are listed in Supplementary Table 1.

#### Biting heterogeneity

Mosquito biting heterogeneity is an important consideration in malaria transmission modelling, and is expected to have a more pronounced impact on *P. vivax*, where a single infective bite can lead to multiple relapses. We varied the standard deviation of the biting heterogeneity distribution such that 80% of bites are received by 20, 40 and 60% of the population (compared with the default of approximately 33% of the population receiving 80% of bites).

#### Treatment coverage

Both equilibrium solutions can incorporate schizont-targeting treatment, defined as the proportion of clinical cases that receive treatment, assumed here to have 100% efficacy. In practice, treatments can be efficacious for both species, though these efficacies are likely to vary, including with levels of pre-existing drug resistance^1,24^. We calculated the impact of varied treatment coverage on the matched equilibrium solutions, assuming that either one or both parasite species was treated at 20, 40 and 60% coverage. The *P. vivax* equilibrium model does not currently include hypnozoite-targeting drugs, which are therefore omitted from this part of the analysis.

#### Mosquito bioparameters

We independently varied the proportion of blood meals that are taken from humans between 0.5 and 0.92, the mosquito biting rate between 1/3 and 2/3, and the adult mosquito death rate between 0.1 and 0.132, for both parasite species.

We also calculated the corresponding equilibrium clinical and total (clinical and sub-clinical) incidence under the matched-mosquito-density assumption, focusing on the relative proportion of cases attributed to each parasite species, disaggregated by age group, across a range of mosquito densities.

#### Country-specific Duffy negativity and relapse rate tailoring

Building on the per-factor analyses above, we constructed a combined model in which Duffy negativity and *P. vivax* relapse rate were set to country-specific values (from MAP^52^ and Battle et al.^61^, respectively), and compared the resulting equilibrium predictions with the MAP co-prevalence estimates for the corresponding subset of countries. This allowed us to assess whether incorporating these biological and demographic factors jointly improves how well the model captures co-prevalence variation.

### Species-specific intervention responses

Building on the equilibria analysis, we next examined species-specific responses to interventions using dynamic simulations. We compared the impacts of two interventions affecting both *P. falciparum* and *P. vivax*: a human-targeted treatment (schizont-targeted drug therapy) and a vector-targeted measure (an ITN distribution) over a range of matched mosquito densities. Simulations were run from equilibrium, using the default parameter sets^29^ for each parasite species, for 20 years consisting of a ten-year burn-in followed either by schizonticidal treatment at 90% coverage for three years (assuming 100% drug adherence), or a single ITN distribution resulting in 90% net usage of the human population, with human population size scaled up to 100,000 at low EIR to avoid complete parasite extinction. The simulations were run with enough time to include the decreasing impacts of the ITN distributions through waning insecticide efficacy (with half-life of 2.5 years) and net loss (assuming an exponentially decreasing net use with average use length of 3 years), and past the removal of drug treatment to demonstrate the species-specific impact of removing those interventions. Twenty stochastic replicate simulations were conducted for each parasite using the default parameter sets.

## Results

### Global parasite prevalence trends

**Fig. 1:**
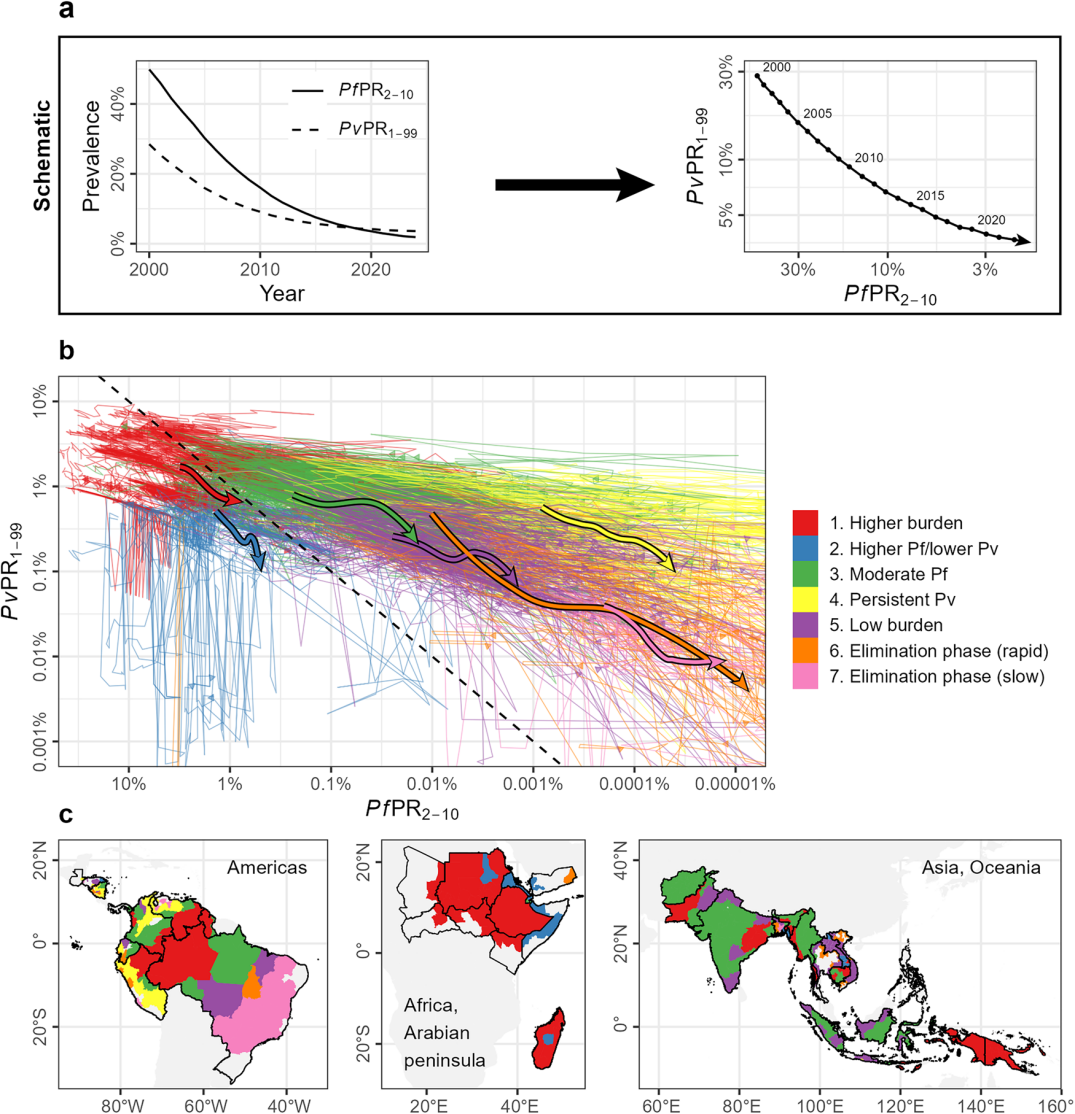
Clustered global admin-level parasite-specific prevalence trends. **a** is a schematic showing how light microscopy prevalence trends of *P. falciparum* (ages 2–10 years: *Pf*PR_2-10_) and *P. vivax* (all ages: *Pv*PR_1–99_) through time from 2000 to 2024 (left panel) are replotted with the parasite prevalences as the *x* and *y* axes (right), where the direction through time towards the present is denoted by the arrowhead. Note that the *x*-axis is reversed such that the low prevalence is on the right-hand side to reflect generally decreasing trends through time. **b** shows these prevalence trends for all admin 1 units, separated into rural and urban regions, with non-zero estimates of both species prevalences at each yearly time point (2000–2024), generated from Malaria Atlas Project estimates. Trajectories are coloured by archetype cluster group using *k*-means clustering with Gap statistic to assign *k*, and descriptive labels assigned to each group. Loess model lines fitted to log_10_ population-at-risk-weighted time-specific averages of each cluster are shown with a bold arrow. The *x* = *y* line is shown with a black dashed line. **c** shows the geographical distribution of the rural clusters.

Yearly *P. falciparum* and *P. vivax* prevalence estimates during 2000–2024 were compiled from the MAP^52^ at the urban/rural subdivision level of admin 1 units. Of these prevalence estimates, we identified 769 urban/rural level regions with non-zero prevalence for both parasites during all examined years, representing 33 countries from Africa, the Eastern Mediterranean, Central and South America, and Southern and South-East Asia (Supplementary Fig. 1). These species-specific prevalence trends are presented jointly (Fig. 1b), with the representation explained schematically in Fig. 1a. Prevalences ranged from 59.5% *P. falciparum* (in Eastern Equatoria, South Sudan in 2000) and 9.2% *P. vivax* (in Mizoram, India in 2012) to near-zero in both species (in various regions and time points), with trends showing a decrease in both estimated parasite prevalences through time between 2000 and 2024 in 563 regions. The average relative decrease in prevalence for each parasite species between 2000 and 2024 was greater in *P. falciparum* (81.8%) than in *P. vivax* (60.5%) in regions where prevalence declined in that parasite species. Accordingly, *P. falciparum* prevalence was lower than *P. vivax* in 75.8% of all co-endemic regions over all timepoints during 2000–2024, a pattern that becomes more pronounced at low *P. falciparum* prevalence. Specifically, *P. falciparum* prevalence < *P. vivax* prevalence in 95.7% of co-prevalence estimates when *P. falciparum* < 0.1%, versus only 14.8% when *P. falciparum* prevalence ≥ 0.1%. The nature of the co-prevalence decreases is, however, highly variable across regions in terms of size and relative changes in parasite prevalence ratios through time (which are broadly shown by the gradient when parasite prevalences are plotted against each other, as shown in Fig. 1b). The remaining 206 urban/rural level regions that did not see an estimated decrease in both parasite species between years 2000–2024 saw an increase in either *P. falciparum* (*n* = 72) or *P. vivax* (*n* = 59) only, or a prevalence increase in both (*n* = 75) regions.

Prevalence trends through time for each region at the urban/rural level were assessed using *k*-means clustering, optimised using the Gap statistic, that partitioned the trends into seven groups (Supplementary Fig. 2). These clusters can serve as broad, archetypical co-endemic trend patterns, which we have labelled descriptively as “*Higher burden*”, “*Higher Pf/lower Pv*”, *“Moderate burden*”, “*Persistent Pv*”, “*Low burden*”, “*Elimination phase (rapid)*” and “*Elimination phase (slow)*”. The parasite co-prevalence trajectories shown in Fig. 1b are coloured according to cluster group, with the geographic spread of rural clustered regions presented at the admin 1 level in Fig. 1c. The broad range of trend values included within each of these clusters is summarised in Table 1.

**Table 1:** Parasite co-prevalence cluster archetypes

		MAP *P. falciparum* prevalence (2–10 years)		MAP *P. vivax* prevalence (1–99 years)	
		Median	95% quantiles	Median	95% quantiles
**Clusters**					
1	Higher burden	3.3%	0.03–30%	1.5%	0.46–5.9%
2	Higher Pf/lower Pv	1.1%	0.02–12%	0.35%	0.0023–1.7%
3	Moderate burden	0.061%	<0.001–1.8%	0.8%	0.15–2.2%
4	Persistent Pv	<0.001%	<0.001–0.082%	0.39%	0.029–1.5%
5	Low burden	0.0079%	<0.001–0.49%	0.2%	0.025–0.84%
6	Elimination phase (rapid)	<0.001%	<0.001–0.27%	0.1%	<0.001–0.62%
7	Elimination phase (slow)	<0.001%	<0.001–0.0029%	0.018%	<0.001–0.31%

Median and 95% quantiles of Malaria Atlas Project (MAP) average co-prevalence value estimates (ages 2–10 for *P. falciparum*, all ages for *P. vivax*), grouped into clusters of co-prevalence trends during 2000–2024 at the urban/rural level division of admin 1 units.

“*Higher burden*” regions include most *P. vivax* endemic regions across Africa, PNG, parts of Asia and the Amazon. These show high *P. falciparum* and *P. vivax* prevalence, with the strongest *P. falciparum* dominance in African regions where transmission is highest. The high prevalence of Duffy negativity limits the *P. vivax* burden in some of these regions, a feature seen more clearly in the “*Higher Pf/lower Pv*” cluster, which includes the remaining African regions and some regions in Yemen. “*Moderate burden*”, “*Low burden*” and “*Elimination phase (slow)*” regions begin at moderate to very low levels of *P. falciparum* and *P. vivax* in 2000 and show steady declines in both species, with *P. falciparum* prevalence typically falling more quickly. The “*Elimination phase (rapid)*” regions begin at similar prevalences to the low burden group, but end at prevalences more similar to the “*Elimination phase (slow)*” cluster, distinguished primarily by a more rapid pace of decline. “*Persistent Pv*” regions also follow a downward trajectory but begin with very low *P. falciparum* prevalence and appreciably higher *P. vivax* prevalence, with the latter persisting at low levels even as *P. falciparum* approaches elimination levels.

Co-prevalence trends subset by country (Supplementary Fig. 3) show differences in the relative species-specific changes, demonstrated with the gradient of a linear model fit. All countries have a parasite ratio gradient less than one, indicating greater variation in *P. falciparum* compared with *P. vivax*, but country-level trends can be steep (indicating similar relative changes in parasite co-prevalences; e.g., with a gradient of 0.99 in Djibouti) or shallow (indicating larger relative changes in *P. falciparum* compared with *P. vivax*; e.g., with a parasite ratio gradient of 0.09 in Afghanistan and Laos). Somalia is unique in having a parasite ratio gradient < 0, indicating a negative relationship between *P. falciparum* and *P. vivax* prevalences, although the estimates from this country are highly variable.

### Model equilibrium comparison analyses

**Fig. 2:**
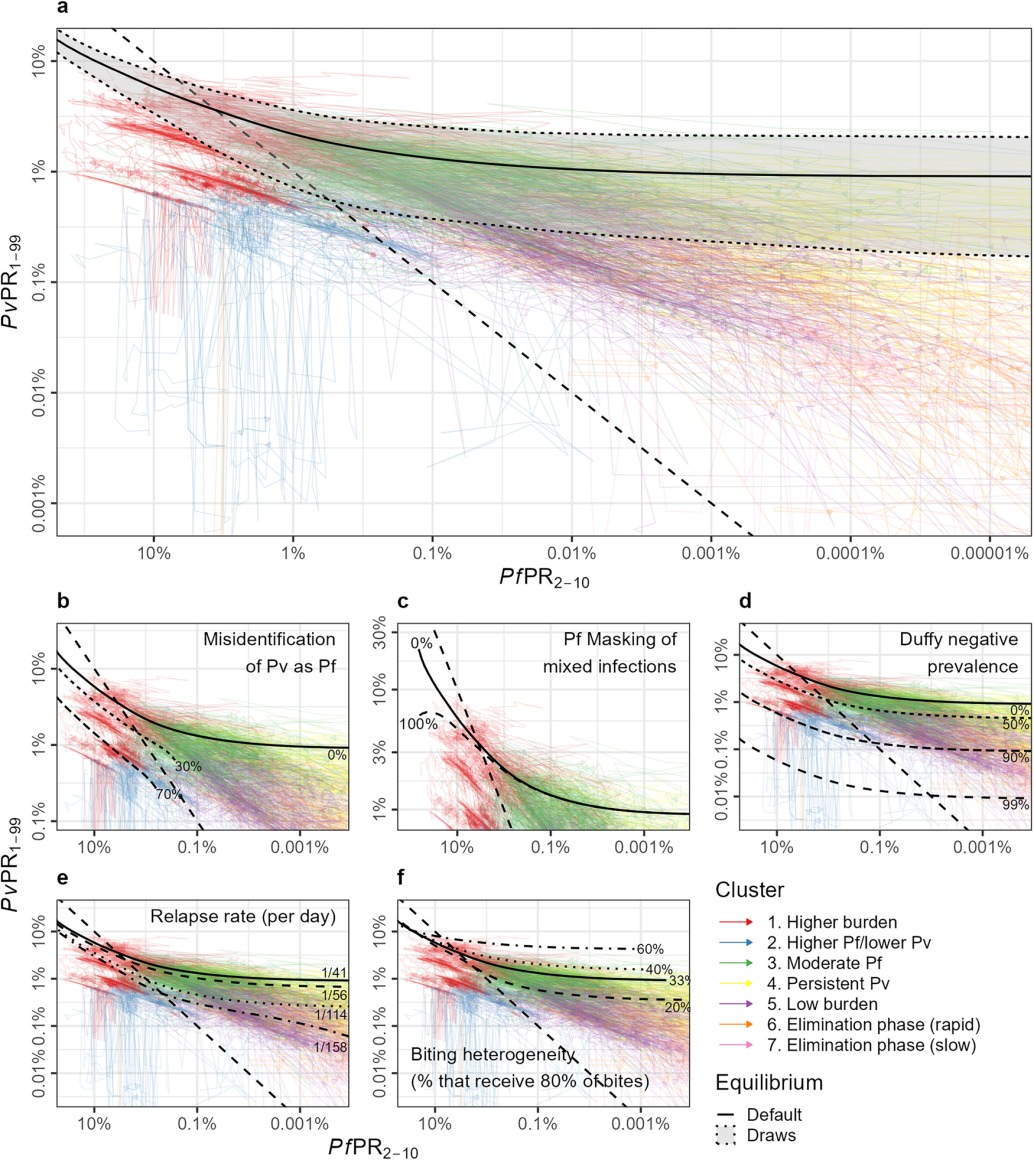
Equilibrium prevalence comparison. **a** Shows the mosquito density matched *P. falciparum* (*Pf*PR_2–10_, ages 2–10) and *P. vivax* (*Pv*PR_1–99_, all ages) model equilibrium solution prevalences under default parameter assumptions (solid black line) with quantile-based uncertainty regions (capturing about 90% of variation) taken from 50 parameter draws (dotted black line and grey ribbon) and shown against Malaria Atlas Project co-prevalence trajectories^52^, coloured by cluster. **b**–**f** Show the default equilibrium under a range of modifying assumptions that impact the relationship between parasite co-prevalences (and where variation in the assumptions is represented by linetype): a percentage of *P. vivax* infections are misidentified as *P. falciparum* (**b**), a percentage of mixed *P. falciparum*-*P. vivax* infections are masked and interpreted as *P. falciparum* (**c**), Duffy negativity prevalence (**d**) assuming Duffy negativity provides 100% protection from infection, variable relapse rate as estimated by Battle et al.^61^ (**e**), and variable mosquito biting heterogeneity (**f**, where each line represents the percentage of the population that receives 80% of mosquito bites, such that 20% represents high heterogeneity and 80% represents no heterogeneity).

In our comparison of the *P. falciparum* and *P. vivax* equilibrium models, we find that the relationships between the EIR, mosquito density and prevalence are non-linear and distinct for each species (Supplementary Fig. 4a–c). Broadly, we can see that *P. falciparum* has higher LM-detectable prevalence than *P. vivax* at higher mosquito density, but that this pattern is reversed at lower densities, where *P. vivax* LM-detectable prevalence is maintained by relapse infections. We also observe a greater gap between PCR- and LM-detectable prevalence for *P. vivax* infections, compared with *P. falciparum* infections, corresponding with the low *P. vivax* parasitaemia and the impact of multiple relapse infections on *P. vivax* immunity that results in lower parasite density. When we compare these model outputs matched by mosquito-density with the MAP prevalence estimates^52^ (Fig. 2a), we observe that the equilibria generally approximate the estimated prevalences, i.e., the equilibrium solutions show greater variation in *P. falciparum* compared with *P. vivax* prevalence as mosquito density changes, to the extent that 51% of the MAP co-prevalence estimates are captured within the equilibria QBURs, although this percentage varies by country trends (Supplementary Fig. 5 and Supplementary Table 1). Trends in some countries are captured well (e.g., Afghanistan captures 99% of estimates), while estimates from some other countries are very poorly captured, including several African countries where none of the estimates are captured and where Duffy negativity is high, and low-density regions such as those in Brazil and Thailand. We observe that the modelled *P. falciparum* prevalence trends toward elimination at a *P. vivax* prevalence of 0.15–2%, with 48% of MAP co-prevalence estimates found with a lower *P. vivax* prevalence than that included within the QBURs.

We find that increasing the level of misidentification of monoparasitic *P. vivax* infections as *P. falciparum* infections shifts the equilibrium relationship towards higher *P. falciparum* and lower *P. vivax* prevalences (Fig. 2b), enabling the capture of previously unaccounted for parasite co-prevalence estimates. A similar pattern can be seen when we model the masking of mixed *P. falciparum*-*P. vivax* infections, which are then misidentified as monoparasitic *P. falciparum* infections (Fig. 2c), although, as expected, the impact only plays a substantial role at higher parasite prevalences where mixed infections (represented by the difference between the two lines in Fig. 2c) are more common.

When we assume that individuals with Duffy negativity have 100% protection from *P. vivax* infection, we observe marked shifts decreasing the *P. vivax* prevalence (Fig. 2d), where a Duffy-negative prevalence of 90% results in a parasite prevalence decrease of an order of magnitude and a prevalence of 99% results in a decrease of two orders of magnitude. Inclusion of country-specific estimates of Duffy-negative prevalences enable the matched model equilibria to account for much more variation in the estimates (61.1% overall, Supplementary Table 1), including previously unaccounted for variation in Africa and the Arabian Peninsula, where countries have high prevalence (> 90%) of Duffy negativity. Duffy negativity in other regions within the African diaspora (e.g., Colombia, Panama, Ecuador, Honduras, Brazil) has a much lower estimated prevalence (< 30%), with correspondingly smaller impacts on malaria prevalence, but which may still be important to consider. These Duffy-negative individuals may also cluster sub-regionally, resulting in substantial heterogeneity in Duffy-negative prevalence and corresponding variation in malaria prevalence.

By calculating the equilibrium solution prevalences over a range of relapse rates estimated by Battle et al.^61^, we find that decreasing the relapse rate decreases the *P. vivax* prevalence, as expected (Fig. 2e). This enables MAP prevalence variation to be better captured in two of the countries from the central Americas, where relapse rate is markedly lower (Honduras and Nicaragua; Supplementary Table 1) and several African countries (Eritrea, Ethiopia, Kenya). Capture of the variation in other countries is made markedly worse with the inclusion of country-specific relapse rates such as Afghanistan, Djibouti, Guatemala, Panama and Sudan, but still captures more variation (60.1%) than the unadjusted equilibrium QBURs.

We see that increasing the biting heterogeneity broadly shifts the equilibrium relationship toward a lower *P. falciparum*-*P. vivax* ratio at lower mosquito densities, and a steeper parasite ratio gradient (Fig. 2f). The equilibrium patterns at higher biting heterogeneity more closely reflect the co-prevalence values seen in the “*low burden*” and “*elimination phase*” clusters from Fig. 1b.

Increasing coverage of schizont-targeting drug treatments in either species decreases the equilibrium prevalence of that species, but we find that the impact of treatments with the same coverage are less pronounced in *P. vivax*, again due to the hypnozoite relapses that are not addressed by schizont-targeting treatments alone (Supplementary Fig. 6a). When the equilibrium co-prevalences are calculated for the scenario where schizont-targeting treatment is applied to both species at equal coverage, we see that the equilibrium solutions do not deviate substantially from the solutions without treatment, albeit with wider variation over parameter draws (Supplementary Fig. 6b).

Varying mosquito bioparameters (biting rate, adult death rate, proportion of human blood meals) had similar impacts on the prevalences of both parasites, and had limited effect on the co-parasite equilibrium relationship (Supplementary Fig. 7a–c).

The combined impact of country-specific estimates of Duffy negativity and relapse rates on the equilibrium solution and its relationship with country subset MAP estimates is shown in Supplementary Fig. 8, with 65.5% of co-prevalence estimates now being captured by QBURs (Supplementary Table 1). This increased capture is particularly well seen in areas of high Duffy negativity (CAR, Chad, Eritrea, Ethiopia, South Sudan and Sudan), and areas where either the change in relapse rate (Central American countries) or the combination of relapse rate change and Duffy negativity increase has improved the fit (Colombia, Ecuador, Guyana, Venezuela), where most of the variation is captured. The model capture of prevalence in some countries, however, was made worse, including in Afghanistan, Djibouti, Guatemala and Panama, and where the changes in relapse rate or the impact of Duffy negativity moved the equilibria *P. vivax* prevalence lower than the prevalence estimates. The variation in some countries, particularly visible in Kenya, shows an improved capture with the inclusion of Duffy negativity that is then made worse by the further inclusion of relapse rates.

Clinical and total (clinical and all asymptomatic infections, including sub-patent or PCR-detectable infections) incidence at the modelled equilibria both show a higher proportion of *P. vivax* infections compared with *P. falciparum* across mosquito densities (Supplementary Fig. 9), consistent with the high rate of relapse infections per infective bite, despite the higher *P. falciparum* prevalence at high mosquito density. This can be reconciled with a shorter duration of *P. vivax* infection. *P. vivax* LM-detectable infections are modelled to last on average about 17 days, and PCR-detectable infections are modelled to last on average 10–50 days (depending on human immunity), while the parallel asymptomatic and sub-patent *P. falciparum* human state infections are modelled to last on average 195 and 110 days, respectively; clinical infections are modelled to last 5 days on average in both parasites^29^. More *P. vivax* infections are therefore required to maintain a given prevalence, relative to *P. falciparum*, all else equal. The age-specific patterns of infection (Supplementary Fig. 9) are greater in *P. vivax* compared with *P. falciparum*, due to the combined impact of *P. vivax* relapses and biting heterogeneity, that trigger and strengthen the immune response multiple times in highly infected individuals. This means that *P. vivax* clinical cases will be more heavily weighted towards younger age groups across mosquito densities, compared with *P. falciparum* cases.

### Species-specific intervention responses

**Fig. 3:**
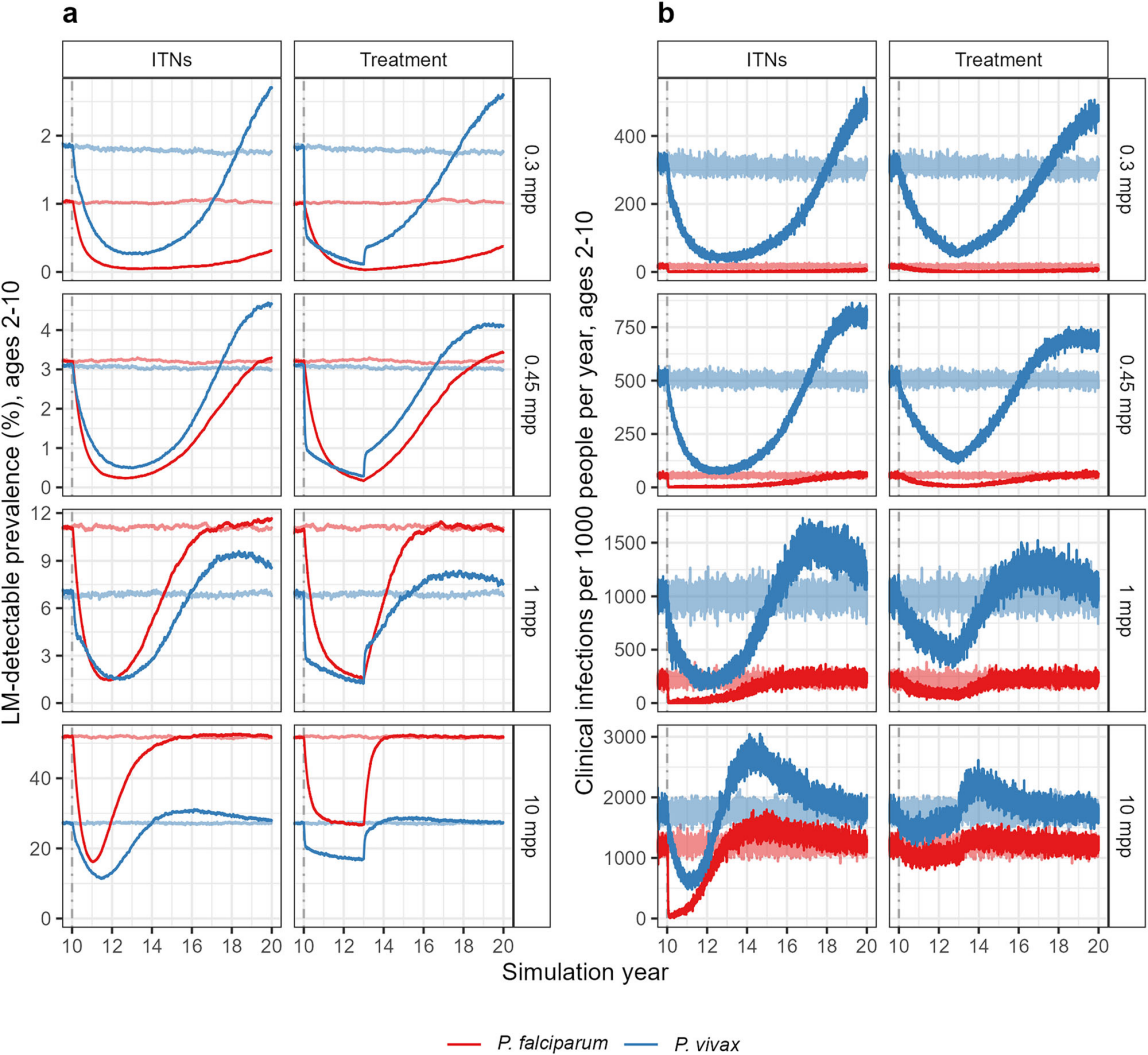
Simulated impact of interventions on species-specific clinical and total infection burden. *P. falciparum* and *P. vivax* model prevalence (**a**, light microscopy (LM) detectable, age 2–10 years) matched by mosquito density from 0.3 to 10 mosquitos per person (mpp; shown across vertical panels). Interventions, either a single insecticide-treated net distribution at 90% population coverage, or a schizont-targeting drug with 90% treatment coverage with 100% efficacy, beginning in year ten (shown with a dot-dashed grey vertical line) and implemented for three years. **b** The clinical incidence results for the same simulations, also for the 2–10 age group. Controls without interventions are shown with translucent lines to identify the baseline prevalence and incidence patterns. The simulations are shown for several years after the interventions are removed.

Figure 3 shows parasite-specific simulation results for two interventions (ITNs and schizont-targeting treatment) and the impact of removing these interventions over a range of EIR values (matched at mosquito density). We see again that *P. falciparum* has a higher baseline LM-detectable prevalence for the 2–10 age group than *P. vivax* at higher mosquito densities, and that this relationship reverses at lower mosquito density, where the prevalences are similar at around 3% prevalence for 0.45 mosquitos per person (Fig. 3a). We also see that the impact of either intervention relative to the baseline prevalence is stronger against *P. falciparum* compared with *P. vivax*, although the absolute impact is greater in the dominant parasite species (*P. falciparum* where *P. falciparum* prevalence > *P. vivax* prevalence, and vice versa). Removing the interventions results in a rebound towards the original baseline prevalence; rebounds are stronger at lower mosquito densities, sometimes exceeding baseline, and are particularly pronounced in *P. vivax*. The parasite-specific impacts and rebounds are due to the continued contribution of relapses from the *P. vivax* hypnozoite reservoir, which are not cleared by these interventions. They are also mediated by transmission-dependent patterns of age-specific immunity. Clinical incidence (Fig. 3b), in contrast, is consistently higher in *P. vivax* than in *P. falciparum* across all mosquito densities (note that the simulation models do not yet account for the protective impact of Duffy negativity), although this difference is less pronounced at higher mosquito density, as previously described in the equilibrium incidence comparison.

The initial impact of clinical treatment on prevalence occurs much more rapidly in *P. vivax* compared with *P. falciparum* (Fig. 3a, Treatment). We have previously noted that the clinical infection rate in *P. vivax* is much higher than *P. falciparum*, due to the contribution of relapse infections. The higher clinical infection rate allows these individuals to receive treatment, removing them from the LM-detectable proportion of the population. The treatment of these individuals greatly reduces the proportion of individuals that would otherwise naturally progress from clinical disease to LM-detectable states, further reducing the observable LM-detectable prevalence. Furthermore, the lower duration of LM-detectable *P. vivax* infections (about 17 days) compared with asymptomatic *P. falciparum* infections (195 days) means that the *P. vivax* infected individuals leave the LM-detectable states more rapidly than LM-detectable *P. falciparum* infections. This high turnover of clinical and LM-detectable infections following the implementation of schizont-targeting treatment induces a faster clearance of infected individuals.

## Discussion

In this paper, we have described a framework that can be used to model *P. vivax* in parallel to *P. falciparum*. In doing so, we integrated White et al.’s *P. vivax* model^47^ into *malariasimulation*^29^, which had previously only simulated transmission of *P. falciparum*, including its full intervention set, and enabled parallel modelling of both species for research and applied use. By exploring *P. falciparum* and *P. vivax* dynamics with a matched-mosquito-density framework, we have shown that these models are able to capture the variation of estimated parasite prevalence patterns in co-endemic regions of the world^52^. Additional factors, such as Duffy negativity and variable hypnozoite relapse rate^61^ were, in many countries, able to improve the capture of the prevalence estimate trends. Others, such as parasite misidentification and mosquito biting heterogeneity were shown to be influential to the modelled relationship but are currently more difficult to quantify. Our work highlights a new opportunity to explore *P. falciparum* and *P. vivax* dynamics within a coordinated modelling framework. However, fully combining the models in practice will depend on accurate estimation of factors such as Duffy negativity, relapse rate, mosquito biting heterogeneity, and the mosquito-species-specific relationships between infection prevalence and mosquito density.

Our simulations highlight important differences in how *P. falciparum* and *P. vivax* respond to interventions. For both *P. falciparum* and *P. vivax*, ITNs and treatment produce sharp declines in prevalence across a wide range of transmission intensities, but these reductions are quickly lost once interventions are withdrawn. Compared with *P. falciparum*, *P. vivax* shows smaller, slower declines and stronger rebounds above the pre-intervention prevalences following the withdrawal of those interventions, consistent with prior findings^47^, reflecting the resilience of *P. vivax* transmission due to relapses. *P. vivax* also shows a more rapid response to schizont-targeting treatment compared with *P. falciparum*, with implications for drug treatment regimens, although whether this finding generalises to real-world settings remains to be determined. At low mosquito densities, *P. falciparum* can be driven to very low prevalences, whereas *P. vivax* persists even under high coverage interventions. These results suggest that while conventional tools are highly effective for *P. falciparum*, additional tools, such as hypnozoite-targeting drug treatments, are required to control *P. vivax*. These findings are consistent with the prevailing understanding that while *P. vivax* is frequently found at lower prevalence than *P. falciparum* in regions of high burden, such as in PNG^62,63^, Ethiopia^64^ and Madagascar^65^, *P. vivax* causes greater burden and poses greater challenges to elimination than *P. falciparum* in low burden and elimination regions^66,67^. A key issue is the identification and treatment of individuals with hypnozoite reservoirs that are currently not directly detectable and who may not have detectable *P. vivax* infections in the blood, but who are likely to experience future relapses. Some indirect indicators of hypnozoite reservoirs include a *P. vivax* blood infection, *P. vivax* seropositivity^9,10^ and even *P. falciparum* infections^68^ (due to heterogeneity in mosquito biting increasing the likelihood of mixed infections^69^), and which can increase treatment of hidden *P. vivax* infections. Ultimately, direct tests to identify hypnozoite infections are needed to more comprehensively address *P. vivax* endemicity.

To facilitate direct comparisons of our model framework results with prevalence trends across many countries, we have opted to use MAP LM-detectable parasite prevalence estimates. Although these estimates are data-driven, drawing on incidence and prevalence surveys together with environmental and intervention data, they remain modelled outputs that depend on their own underlying assumptions and uncertainties, including parasite-specific incidence-prevalence relationships that are variable by country. The limits of these estimates are apparent from the small number of sub-Saharan African countries where MAP estimates a non-zero presence of *P. vivax*, in contrast to the large number of countries where *P. vivax* has been identified despite the high prevalence of Duffy negativity^16^. Furthermore, the parasite-specific prevalences are estimated for different age groups, in accordance with the majority of available prevalence data (2–10 years for *P. falciparum* and 1–99 years for *P. vivax*), such that comparisons between these will not be entirely representative of comparative burden. Prevalences in both species are likely to be higher in younger age groups due to the increase in historic-infection-induced immunity in older age groups, a pattern that is stronger in *P. vivax* due to relapses. Future work may involve tailoring and calibration of the transmission model framework to country- and age-specific parasite prevalence data.

While the *P. falciparum* and *P. vivax* model framework shown here is a step forward for malaria modelling in co-endemic regions, there are several limitations which represent opportunities for future model development. Primarily, the parallel models do not yet interact with each other, preventing the explicit capture of mixed parasite infections and any potential within-host parasite interactions (human or mosquito), and cross-species intervention effects (e.g., treatment of one species affecting the other) as found in other models^34,38^. The analyses involving treatment coverage should therefore be treated as investigating the relative parasite-specific impact of treatment in the absence of treatment in the other, rather than asserting implications for treatment coverage of one parasite on the prevalence of the other in co-endemic settings. A fully integrated model would enable exploration of the relationship between mosquito biting heterogeneity and the prevalence of mixed parasite infections, providing insight into difficult-to-estimate model parameters. Although the matched model outputs align well with global prevalence estimates, further work is needed to validate the dual calibration of the parasite models using real, time-varying country data. Matching these models by mosquito density is a strong assumption, given that real transmission is mediated by complex, parasite-specific mosquito species mixes; a fuller treatment lies outside the scope of this work. Our framework is generally not appropriate for modelling many co-endemic regions with low prevalence of one or both parasite species due to spatial heterogeneity. Modelling these regions may require features that are not included here, such as explicit spatial structure and human movement. Despite these limitations, the integration achieved here provides a strong foundation for incorporating many of these elements in future work.

Another future priority will be to enhance the model to include additional biologically plausible features, a key area being the role of Duffy negativity in shaping *P. vivax* transmission. Although Duffy negativity does not appear to provide complete protection against infection^13^, the mechanisms by which infection occurs in Duffy-negative individuals remain uncertain and may involve susceptibility in a subset of Duffy-negative individuals, partial susceptibility to some infectious bites, or low-density infections that are rarely detected but still contribute to transmission. Identifying and incorporating key processes mechanistically into the model would improve realism, though it may be possible in some applications to approximate their effects through reduced transmission potential. Addressing these uncertainties will strengthen the model’s applicability to a wider range of epidemiological contexts.

By integrating *P. vivax* dynamics into an established open-source *P. falciparum* modelling platform, alongside its suite of helper packages, this work provides a practical tool for exploring species-specific intervention effects in co-endemic settings that enables a range of different analyses. While developed primarily to compare and tailor models for research questions, the shared framework and intervention toolkit mean it can also be applied directly to support decision-making. As more detailed data on species interactions, Duffy negativity, relapse patterns and biting heterogeneity become available, the models in this framework can be refined to better reflect local epidemiology. In turn, this will enable more accurate forecasts of how interventions shape the relative burden of each species and the relative contribution of *P. vivax* relapses versus infective bites to transmission, helping to design control and elimination strategies that remain effective as *P. falciparum* declines and *P. vivax* becomes increasingly dominant: a critical step towards sustainable malaria elimination in diverse transmission settings.

## Supplementary information

Supplementary Information

## Data Availability

The data analysed in this study are publicly available from external sources and were not generated by the authors. Yearly first-administrative-level estimates of *P. falciparum* and *P. vivax* light microscopy-detectable parasite prevalence (2000–2024), country-level Duffy negativity prevalences, and the transmission masks used to define populations at risk were obtained from the Malaria Atlas Project (https://malariaatlas.org/) and are programmatically accessible through the *malariaAtlas* R package (https://CRAN.R-project.org/package=malariaAtlas). Pixel-level total population estimates and 1-year age-band disaggregations were obtained from the WorldPop “Unconstrained individual countries 2000–2020 UN adjusted (1 km resolution)” rasters (https://www.worldpop.org/). Population projections to 2050 and country age-structure demography were derived from the United Nations *World Population Prospects and World Urbanization Prospects* (https://population.un.org/wpp/, https://population.un.org/wup/). These population sources, together with the urban–rural classification (defined within administrative units using a threshold density of 1500 people per square km) and the age-specific populations at risk for each parasite, are aggregated and made available for the analyses presented here through the *site* R package (https://github.com/mrc-ide/site). Country-level estimates of *P. vivax* hypnozoite batch relapse rate were obtained from Battle et al., 2014 (10.1186/1475-2875-13-144). Further information is available from the corresponding author (R.J.S.) on request.

## Code availability

All code required to reproduce the analyses presented in this study, together with instructions for use, is openly available at https://github.com/RJSheppard/pfpvmodelcomparison and is archived on Zenodo (10.5281/zenodo.17700498). The analyses were performed in R using the *malariasimulation* package (https://github.com/mrc-ide/malariasimulation) and the *site* R package (https://github.com/mrc-ide/site), which is part of the broader *malariaverse* package set (https://mrc-ide.github.io/malariaverse/).
