# Supplementary material for: Integrating parallel Plasmodium falciparum and Plasmodium vivax malaria models in a unified framework to capture co-endemic prevalence patterns
Source: Commun Health. 2026 Jul 7;1(1):11. doi: 10.1038/s44528-026-00007-4 (PMC13341314; doi:10.1038/s44528-026-00007-4)
Supplement: Supplementary file 1 — Supplementary Information [file 44528_2026_7_MOESM1_ESM.pdf]

Supplementary Information

Integrating parallel *Plasmodium falciparum* and *Plasmodium vivax* malaria models in a unified framework to capture co-endemic prevalence patterns

Richard J. Sheppard[1*], Giovanni D. Charles[1], Constanze Ciavarella[2], Nora Schmit[1], Shazia N. Ruybal-Pesántez[1,3], Gina Cuomo-Dannenburg[1,4], Tom R. Brewer[1], Michael T. White[5], Peter Winskill[1].

1. MRC Centre for Global Infectious Disease Analysis, Department of Infectious Disease Epidemiology, Imperial College, London, UK

2. PCCEI, Université de Montpellier, INSERM, Université des Antilles, Montpellier, France

3. Instituto de Microbiología, Universidad San Francisco de Quito, Quito, Ecuador

4. Department of Microbiology and Immunology, Rega Institute, KU Leuven -- University of Leuven, Leuven, Belgium

5. Infectious Disease Epidemiology and Analytics G5 Unit, Department of Global Health, Institut Pasteur, Université Paris-Cité, INSERM U1347, Paris, France

* Corresponding author

Supplementary Figures

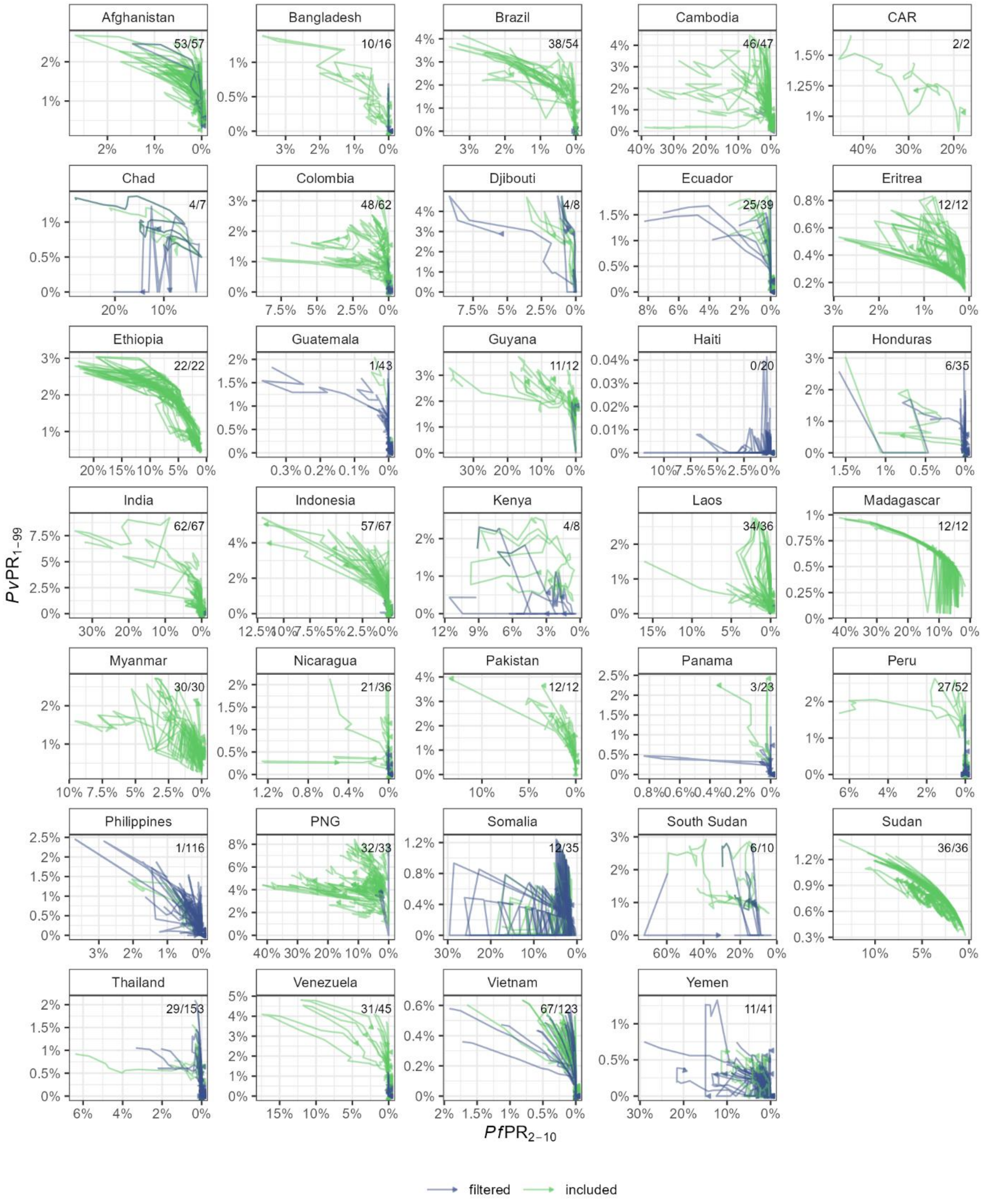

Supplementary Figure 1 | All prevalence trajectories. Yearly *Plasmodium falciparum* prevalence (ages 2-10, $PfPR_{2\text{-}10}$) plotted against *P. vivax* prevalence (ages 1-99, $PvPR_{1\text{-}99}$) where lines show how the co-prevalence trends change

through time from 2000-2024. Regions are plotted at the admin 1, urban/rural level for each region where both species are found in at least one timepoint, as estimated by the Malaria Atlas Project[1]. Trajectories are coloured by whether the trajectory was used in cluster analysis or filtered (dependent on whether any timepoint was equal to 0, prohibiting it from being used in the analysis), and facetted by country, with the number of remaining trajectories out of the total given in the top right of each panel.

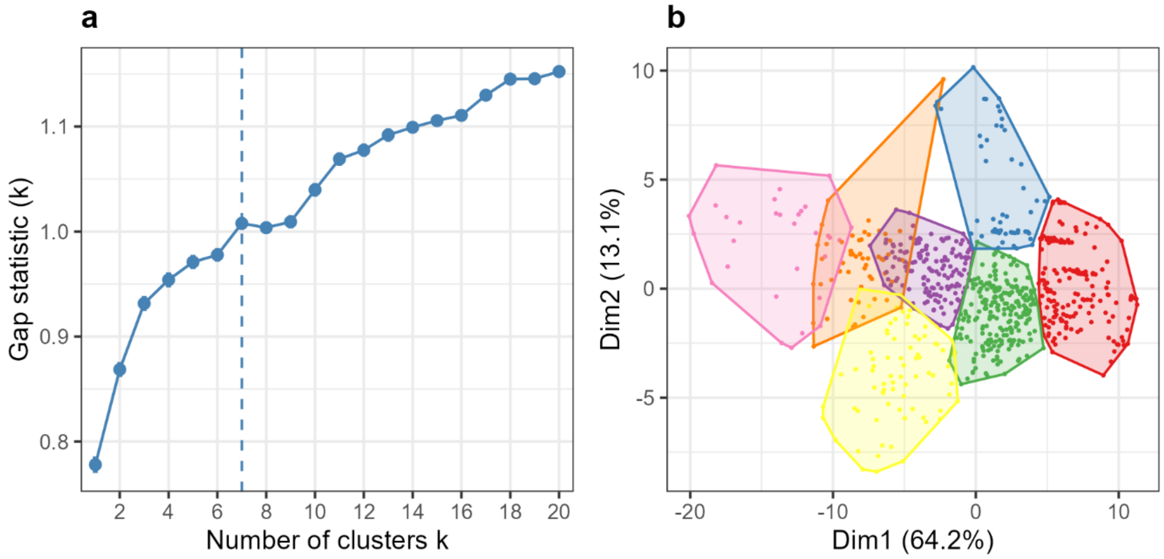

Supplementary Figure 2 | Gap cluster number optimisation. (**a**) Gap statistics are shown against the number of clusters from *k*-means clustering of Malaria Atlas Project[1] *P. falciparum* and *P. vivax* co-prevalence estimates from 2000-2024, with (**b**) showing the corresponding principal component plot clusters where *k*=7 and colours represent clusters that correspond with the cluster colours used throughout.

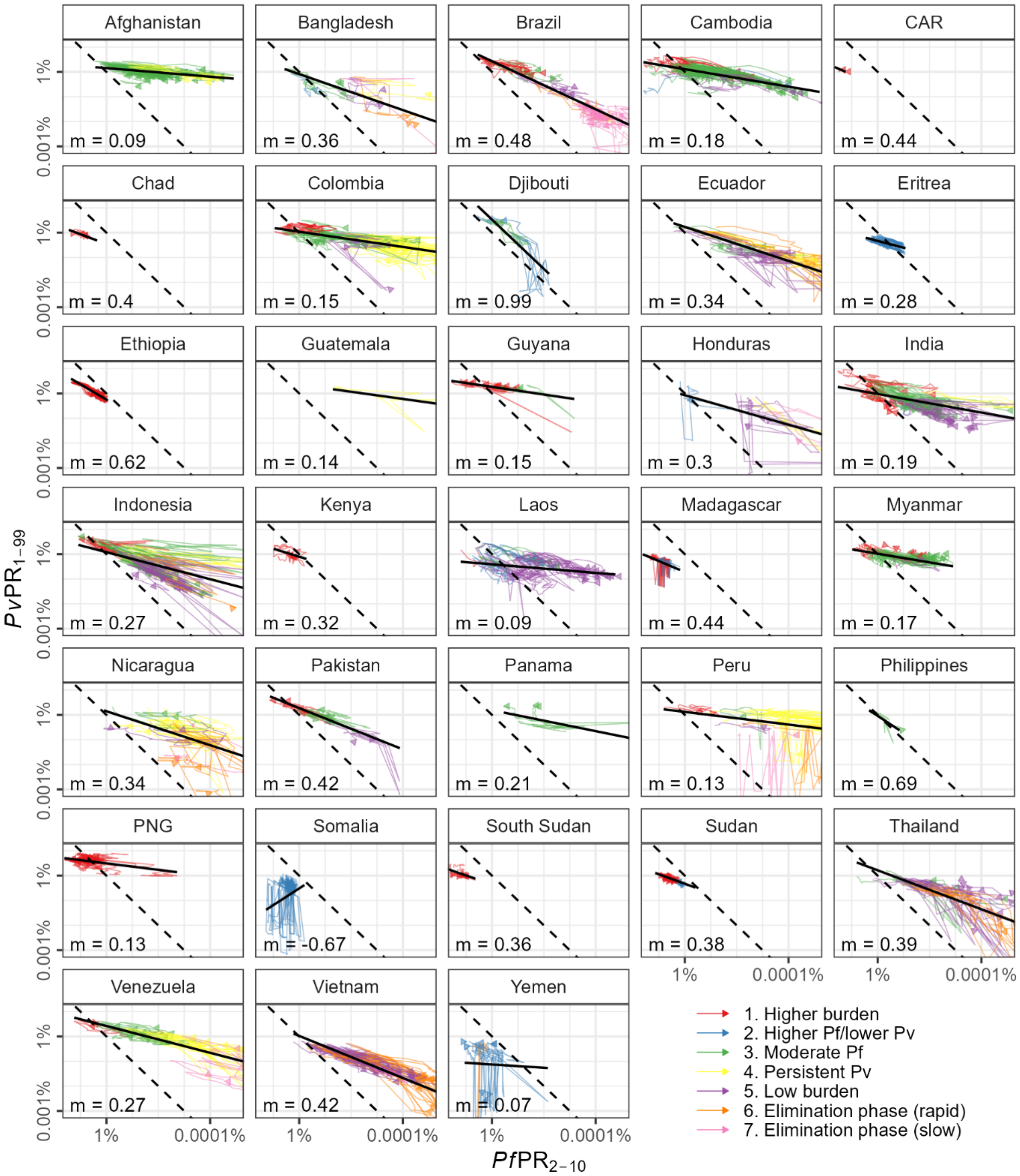

Supplementary Figure 3 | Country subsets of clustered parasite-specific prevalence trends. Malaria Atlas Project [1] *P. falciparum* (ages 2-10 years: $Pf\mathrm{PR}_{2\text{-}10}$) and *P. vivax* (all ages: $Pv\mathrm{PR}_{1\text{-}99}$) light microscopy prevalence trends for all rural and urban regions at the admin 1 unit level with non-zero estimates of both species prevalences at each yearly time point (2000-2024) subset by country. Trajectories are coloured by archetype cluster group, with the direction through time towards the

present is denoted by the arrowhead. The x=y line is shown with a black dashed line while the solid black line shows a country-level linear fit to the prevalence point estimates with the gradient of this line (m) given in the bottom left corner of each panel. Some country names are initialised: Central African Republic (CAR) and Papua New Guinea (PNG).

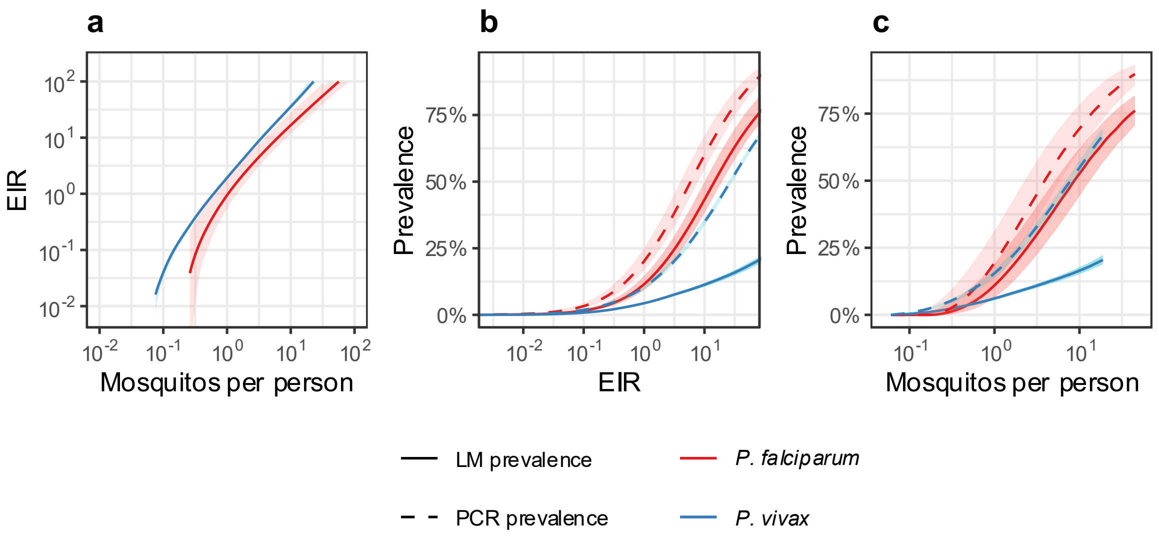

Supplementary Figure 4 | The parasite species-specific equilibrium relationships between EIR, prevalence and mosquito density. (**a**) shows the relationship between entomological inoculation rate (EIR) and mosquito density (mosquitos per person). (**b**) shows the relationships between EIR and human prevalence by modelled light-microscopy detection (LM prevalence) and by polymerase chain reaction (PCR prevalence). *P. falciparum* prevalence is shown for the 2-10 age group (*Pf*PR$_{2\text{-}10}$) while *P. vivax* prevalence is shown for the 1-99 age group (*Pv*PR$_{1\text{-}99}$). (**c**) shows the corresponding relationships between the LM and PCR prevalences and mosquito density (mpp). Lines and ribbons show equilibrium

results for the default parameter set and quantiles over 50 posterior parameter draw sets, respectively.

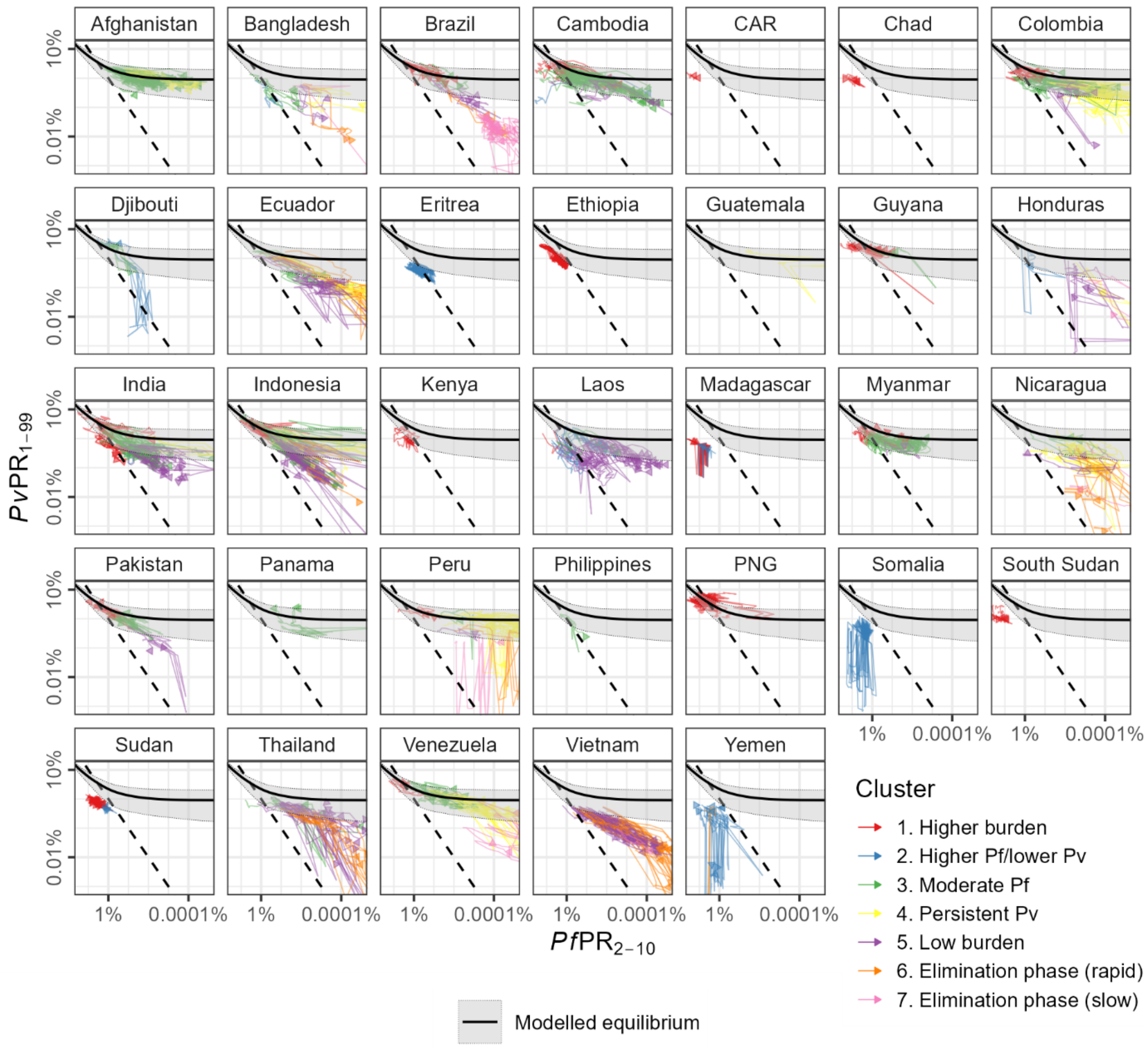

Supplementary Figure 5 | Unadjusted equilibrium prevalence by country. The mosquito density matched *P. falciparum* (*Pf*PR$_{2-10}$, ages 2-10) and *P. vivax* (*Pv*PR$_{1-99}$, all ages*)* model equilibrium solutions under default parameter assumptions with quantile-based uncertainty regions (capturing about 90% of variation, see SI: Supplementary Methods) taken from 50 parameter draws and shown against Malaria Atlas Project[1] prevalence trajectories[1], coloured by cluster and facetted by country.

The *P. falciparum* = *P. vivax* relationship is shown with a dashed black line on each panel.

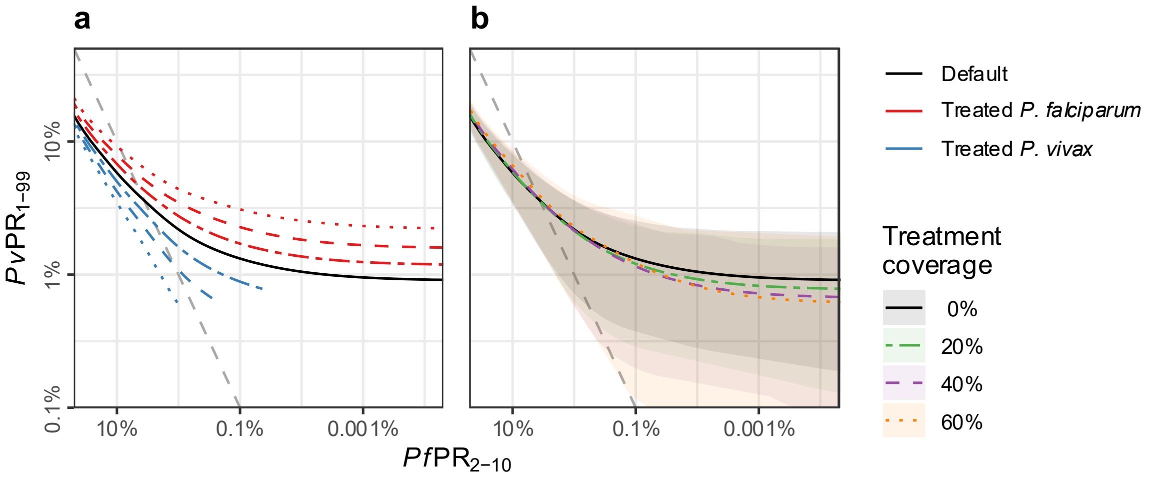

Supplementary Figure 6 | Drug impact on parasite-specific equilibrium prevalence. The impact of varying the coverage of a hypothetical drug treatment with 100% efficacy on equilibrium *P. falciparum* and *P. vivax* prevalence (*Pf*Pr$_{2\text{-}10}$ and *Pv*Pr$_{1\text{-}99}$) where (**a**) treatment is applied to only one species (*P. falciparum* or *P. vivax*) at a time and (**b**) treatment coverage is applied to both species. The *Pf*Pr$_{2\text{-}10}$ = *Pv*Pr$_{1\text{-}99}$ line is shown for reference (grey dashed). Lines and ribbons show equilibrium results for the default parameter set and quantile-based uncertainty regions over 50 posterior parameter draw sets, respectively.

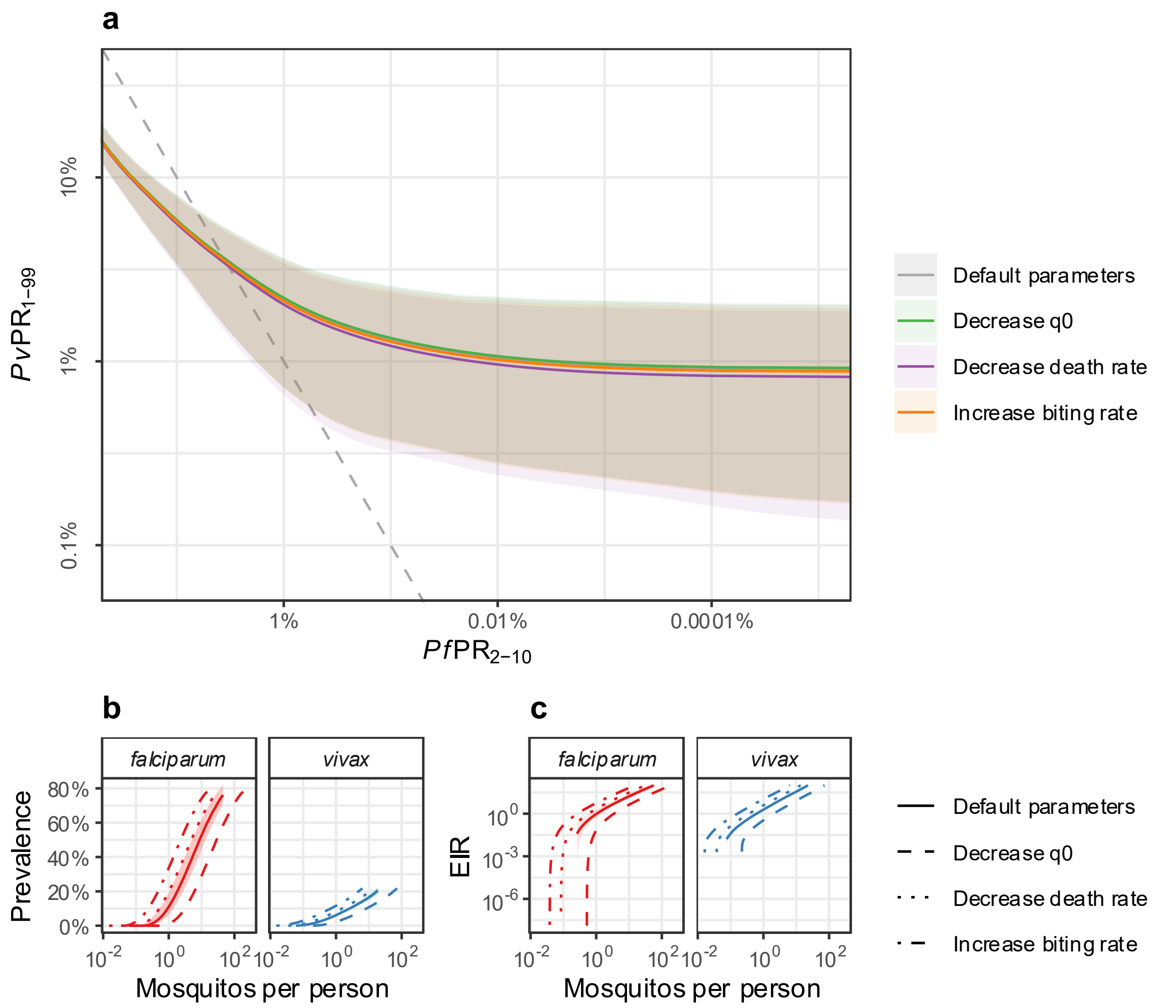

Supplementary Figure 7 | Mosquito parameter impact on parasite-specific equilibrium prevalence. The impact of varying the mosquito bionomic parameters (the proportion of human blood meals (q0), the mosquito death rate and the mosquito biting rate) on equilibrium light-microscopy detectable *P. falciparum* and *P. vivax* prevalence (*Pf*Pr$_{2\text{-}10}$ and *Pv*Pr$_{1\text{-}99}$, panel **a**), with default parameters q0 = 0.9, mosquito death rate = 0.132 and mosquito biting rate = 1/3. This relationship is shown against equilibria where q0 is decreased from 0.9 to 0.5, the mosquito death rate is decreased from 0.132 to 0.1, or the biting rate is increased from 1/3 to 2/3. The *Pf*Pr$_{2\text{-}10}$ = *Pv*Pr$_{1\text{-}99}$ line is shown for reference (grey dashed). Panels (**b**) and (**c**)

show the species-specific relationships between mosquito density (mpp) and light microscopy detectable prevalence, (**b**) and mosquito density and entomological inoculation rate (EIR, **c**) under these bionomic changes for ages 2-10 for *P. falciparum* and ages 1-99 for *P. vivax*. Lines and ribbons show equilibrium results for the default parameter set and quantile-based uncertainty regions over 50 posterior parameter draw sets, respectively.

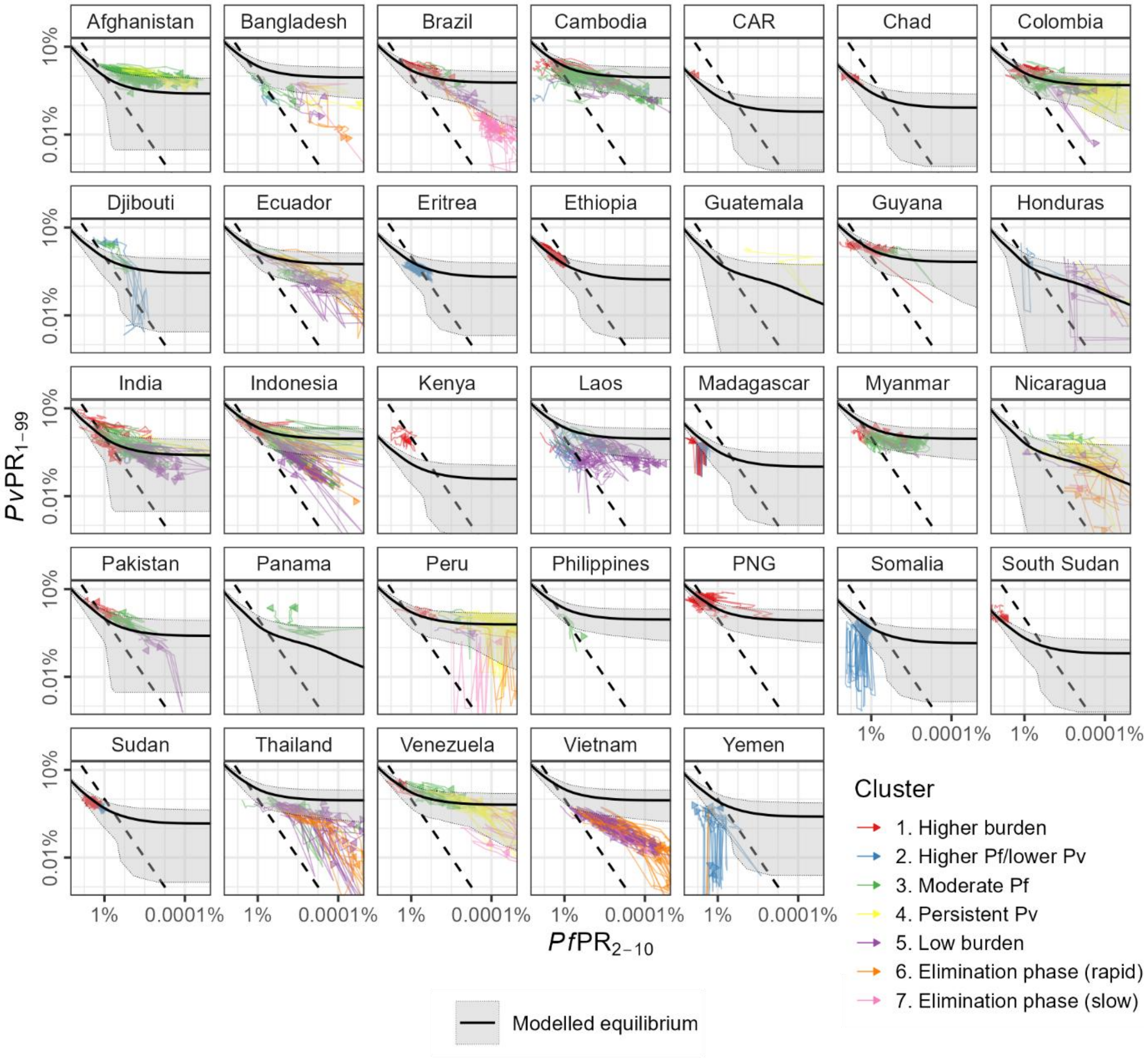

Supplementary Figure 8 | Adjusted equilibrium prevalence by country. The mosquito density matched *P. falciparum* (*Pf*$PR_{2\text{-}10}$, ages 2-10) and *P. vivax* (*Pv*$PR_{1\text{-}99}$,

all ages) model equilibrium solutions under default parameter assumptions, accounting for country estimates of mean Duffy negativity prevalence as estimated by MAP[1] and relapse rates estimated by Battle et al. [2], with quantile-based uncertainty regions (capturing about 90% of variation) taken from 50 parameter draws and shown against Malaria Atlas Project[1] prevalence trajectories[1], coloured by cluster and facetted by country. The *P. falciparum* = *P. vivax* relationship is shown with a dashed black line on each panel.

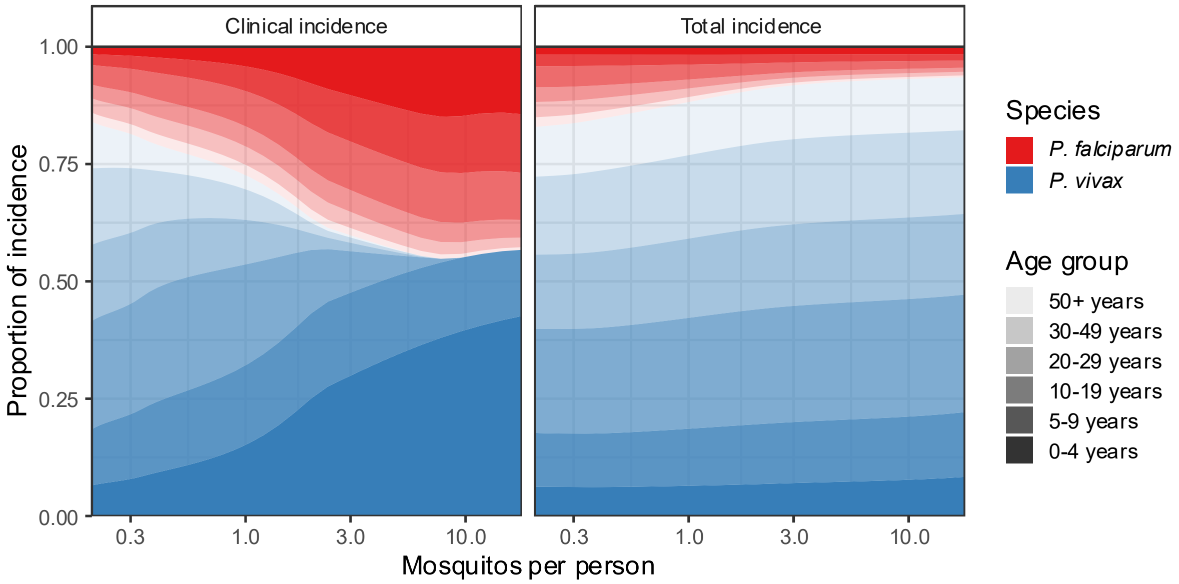

Supplementary Figure 9 | Equilibrium age-structured incidence. Clinical and total (clinical and non-clinical infection, inclusive of LM and PCR-detectable infections) incidence shown over a range of mosquito densities (mosquitos per person) with results shown by age and parasite species. Note that parasite-specific age groups are reflected in the panels, such that youngest age groups are at the top and bottom of the panels and oldest age groups are in the centre,

Supplementary Tables

Country	n	Duffy negativity prevalence (%)	Relapse region	Relapse rate range (days)	Modelled Relapse rate (days)	Points included (%)			
						Default	With Duffy negativity	With relapse rates	With Duffy negativity and relapse rates
Afghanistan	1325	0.6	South Asia	108-120	114	99	99	54.3	53.3
Bangladesh	250	0.5	South-East Asia	41	41	30.4	30.4	30.4	30.4
Brazil	950	10.5	South America	48-65	56	35.7	35.7	38.5	38.6
CAR	50	84.2	sub-Saharan Africa	66-107	86	0	58	0	100
Cambodia	1150	0	South-East Asia	41	41	82.4	82.4	82.4	82.4
Chad	100	78.2	sub-Saharan Africa	66-107	86	0	78	0	97
Colombia	1200	26.8	South America	48-65	56	77.9	88.7	92.3	92.8
Djibouti	100	26.6	sub-Saharan Africa	66-107	86	73	62	64	60
Ecuador	625	15.2	South America	48-65	56	38.2	44	64.8	75.5
Eritrea	300	46.3	sub-Saharan Africa	66-107	86	15.7	77.3	98.7	100
Ethiopia	550	56.3	sub-Saharan Africa	66-107	86	10.4	98.9	86.9	83.6
Guatemala	25	7.6	Central America	152-164	158	96	92	52	44
Guyana	275	0	South America	48-65	56	75.6	75.6	81.5	81.5

Country	n	Duffy negativity prevalence (%)	Relapse region	Relapse rate range (days)	Modelled Relapse rate (days)	Points included (%)			
						Default	With Duffy negativity	With relapse rates	With Duffy negativity and relapse rates
Honduras	150	12	Central America	152-164	158	38	40	90.7	89.3
India	1550	0.9	South Asia	108-120	114	62.7	62.8	85.4	85.1
Indonesia	1425	0.1	South-East Asia	41	41	65.5	65.5	65.5	65.5
Kenya	100	90	sub-Saharan Africa	66-107	86	31	6	80	0
Laos	850	0	South-East Asia	41	41	37.1	37.1	37.1	37.1
Madagascar	300	73.4	sub-Saharan Africa	66-107	86	0	9.7	0	64
Myanmar	750	0	South-East Asia	41	41	86.1	86.1	86.1	86.1
Nicaragua	525	5	Central America	152-164	158	34.3	36	91.4	90.5
PNG	800	0	Oceania	42-47	44	63.7	63.7	61.3	61.3
Pakistan	300	0.8	South Asia	108-120	114	83	83	85	84.3
Panama	75	17.4	Central America	152-164	158	93.3	89.3	70.7	56
Peru	675	7.5	South America	48-65	56	83.7	84.1	88	86.5
Philippines	25	0	South-East Asia	41	41	60	60	60	60
Somalia	300	62.6	sub-Saharan Africa	66-107	86	0	23.7	14.7	56.7

Country	n	Duffy negativity prevalence (%)	Relapse region	Relapse rate range (days)	Modelled Relapse rate (days)	Points included (%)			
						Default	With Duffy negativity	With relapse rates	With Duffy negativity and relapse rates
South Sudan	150	83.4	sub-Saharan Africa	66-107	86	0	59.3	2	66.7
Sudan	900	61.8	sub-Saharan Africa	66-107	86	0	89.7	44.3	100
Thailand	725	0	South-East Asia	41	41	43.6	43.6	43.6	43.6
Venezuela	775	3.3	South America	48-65	56	76.4	77.2	81.5	81.2
Vietnam	1675	0.3	South-East Asia	41	41	6.4	6.4	6.4	6.4
Yemen	275	34.1	sub-Saharan Africa	66-107	86	0	1.5	16	30.2
Global	19225					51.1	61.1	60.1	65.5

Supplementary Table 1 | Country-specific equilibrium parasite prevalence capture. This table shows the country-specific information used to calculate the capture of Malaria Atlas Project (MAP)[1] prevalence estimates of *P. falciparum* (ages 2-10) and *P. vivax* (ages 1-99) by the equilibrium quantile-based uncertainty regions (QBURs; which capture about 90% of equilibrium co-prevalence variation). Number of MAP yearly co-prevalence estimates (2000-2024) for each country are shown with average country-level Duffy negativity prevalence (based on MAP estimates) and relapse ranges from Battle et al.,[2]. The capture of points by QBURs is shown using default assumptions, and taking into account Duffy-negativity, the mean relapse rate within each range

and both Duffy negativity and chosen relapse rates. A summary of the global capture of points using these methods is also given. Cells with capture of MAP co-prevalence was coloured according to the variation capture, with green cells showing better capture and red showing poorer capture.

Supplementary Information References

1. MAP – Malaria Atlas Project. https://malariaatlas.org/ (2025).

2. Battle, K. E. *et al*. Geographical variation in Plasmodium vivax relapse. *Malar. J.* **13**, 1–16 (2014).
